# The Cotton GhWRKY91 Transcription Factor Mediates Leaf Senescence and Responses to Drought Stress in Transgenic *Arabidopsis thaliana*

**DOI:** 10.3389/fpls.2019.01352

**Published:** 2019-10-29

**Authors:** Lijiao Gu, Qiang Ma, Chi Zhang, Congcong Wang, Hengling Wei, Hantao Wang, Shuxun Yu

**Affiliations:** State Key Laboratory of Cotton Biology, Institute of Cotton Research of CAAS, Anyang, China

**Keywords:** GhWRKY91, leaf senescence, abscisic acid, drought, *GhWRKY17*, cotton

## Abstract

WRKY transcription factors (TFs) play essential roles in the plant response to leaf senescence and abiotic stress. However, the WRKY TFs involved in leaf senescence and stress tolerance in cotton (*Gossypium hirsutum* L.) are still largely unknown. In this study, a WRKY gene, *GhWRKY91*, was isolated and thoroughly characterized. Transcriptional activity assays showed that GhWRKY91 could activate transcription in yeast. The expression pattern of *GhWRKY91* during leaf senescence, and in response to abscisic acid (ABA) and drought stress was evaluated. β-Glucuronidase (GUS) activity driven by the *GhWRKY91* promoter in transgenic *Arabidopsis* was reduced upon exposure to ABA and drought treatments. Constitutive expression of *GhWRKY91* in *Arabidopsis* delayed natural leaf senescence. *GhWRKY91* transgenic plants exhibited increased drought tolerance and presented delayed drought-induced leaf senescence, as accompanied by reinforced expression of stress-related genes and attenuated expression of senescence-associated genes (SAGs). Yeast one-hybrid (Y1H) assays and electrophoretic mobility shift assays (EMSAs) revealed that GhWRKY91 directly targets *GhWRKY17*, a gene associated with ABA signals and reactive oxygen species (ROS) production. A transient dual-luciferase reporter assay demonstrated that GhWRKY91 activated the expression of *GhWRKY17*. Our results suggest that GhWRKY91 might negatively regulate natural and stress-induced leaf senescence and provide a foundation for further functional studies on leaf senescence and the stress response in cotton.

## Introduction

The WRKY gene family is a large family of plant-specific transcription factors (TFs) that play important roles in various processes, such as stem elongation (Zhang et al., 2011), pathogen resistance (Pan and Jiang, 2014), leaf senescence (Xie et al., 2014), panicle development (Xiang et al., 2017), trichome and seed coat development (Johnson et al., 2002), pollen development (Guan et al., 2014), fruit ripening (Jiang et al., 2017), and biotic and abiotic stress responses (Pan and Jiang, 2014). WRKY TFs have one or two conserved WRKY domains that consist of approximately 60 amino acids, with a WRKYGQK sequence in the N-terminal region and a C2H2 or C2HC zinc finger motif in the C-terminal region. WRKY TFs can be divided into three groups [I, II (IIa-e), and III] on the basis of their structure and evolutionary history (Eulgem et al., 2000; Rushton et al., 2010). WRKY TFs can interact with W-box cis-elements [TTGAC(C/T)] in the promoter regions of downstream genes to regulate the expression of those genes, thus leading to improved plant adaptation to environmental changes (Fan et al., 2017).

In agricultural production, leaf senescence leads to a decline in photosynthesis, which greatly limits the yield potential of crops. As the final stage of plant development, leaf senescence causes changes in plant cell structure, physiological and biochemical parameters, hormone levels, and gene expression (Lim et al., 2007). Genes expression significantly increases during leaf senescence include those that encode TFs such NACs (NAM, ATAF1/2 and CUC2) and WRKYs (Balazadeh et al., 2008; Guo et al., 2010; Lin et al., 2015; Wu et al., 2016). For example, an aging upregulated NAC TF, GhNAP, delays leaf senescence in RNA interference (RNAi) cotton plants *via* abscisic acid (ABA)-mediated pathways (Fan et al., 2015). A growing number of WRKY TFs are considered senescence regulators in plants (Kim et al., 2017). In *Arabidopsis*, AtWRKY6 (Robatzek and Somssich, 2002; Zhang et al., 2018), AtWRKY22 (Zhou et al., 2011), AtWRKY45 (Chen et al., 2017), AtWRKY53 (Miao et al., 2004), and AtWRKY75 (Guo et al., 2017a) act as positive regulators during natural leaf senescence, whereas AtWRKY18 (Potschin et al., 2014), AtWRKY54 (Besseau et al., 2012), AtWRKY57 (Jiang et al., 2014), and AtWRKY70 (Ulker et al., 2007) function as negative regulators. Furthermore, AtWRKY22 has been shown to promote and delay leaf senescence in overexpression plants and in T-DNA insertion mutants under dark conditions, respectively (Zhou et al., 2011). *Atwrky57* mutants exhibit a jasmonic acid (JA)-induced early leaf senescence phenotype in which auxin functions as an antagonist (Jiang et al., 2014). In rice, OsWRKY23 has been shown to be involved in dark-induced leaf senescence in transgenic *Arabidopsis* plants (Jing et al., 2009). WRKY TFs are also involved in vegetative and fruit senescence, including BrWRKY65 in Chinese flowering cabbage (Fan et al., 2017) and LcWRKY1 in litchi (Jiang et al., 2017). Two cotton WRKY TFs, GhWRKY27 (Gu et al., 2019) and GhWRKY42 (Gu et al., 2018b), were recently identified to promote leaf senescence in transgenic *Arabidopsis* plants, suggesting that WRKY TFs have important roles during leaf senescence in cotton.

Drought or water shortage is one of the main environmental factors that reduces crop yields (Cominelli and Tonelli, 2010). Due to global warming and water shortages, water for crop irrigation is becoming increasingly limited and the development of drought-resistant crop species is particularly important (White et al., 2004). The mechanism of drought resistance in plants is very complex and is usually regulated by multiple genes, including those that encode WRKY TFs (Reynolds and Tuberosa, 2008; Pinto et al., 2010; Dou et al., 2014). Previous reports have shown that overexpression of wheat (*Triticum aestivum* L.) TaWRKY10 confers drought tolerance to transgenic tobacco plants by mediating osmotic balance, the production of reactive oxygen species (ROS), and the expression of stress-responsive genes (Wang et al., 2013). TaWRKY44 positively regulates drought stress in transgenic tobacco plants by scavenging ROS that have accumulated *via* cellular antioxidant systems or stress-related gene expression (Wang et al., 2015). ABA is an important plant hormone that participates in a variety of signal transduction pathways, especially those involved in plant resistance to adverse environmental stimuli such as drought, salt, and low temperature (Cutler et al., 2010). Adverse environmental conditions can cause the rapid accumulation of ABA, thus leading to stomatal closure and reduced water loss, which together constitute the main factor that leads to drought tolerance in plants (Cominelli and Tonelli, 2010; Krasensky and Jonak, 2012). Overexpression of GhWRKY27a reduces the drought tolerance of transgenic tobacco plants, and this effect is associated with enhanced stomatal opening and attenuated expression of ABA- and drought-associated genes (Yan et al., 2015). The constitutive expression of GhWRKY41 in tobacco improves salt and drought tolerance by enhancing stomatal closure in an ABA-dependent manner (Chu et al., 2015). Together, the results of these studies suggest that WRKY TFs play important roles in the drought stress response mediated by ABA signalling.

Plant growth and crop productivity are severely influenced by external environmental factors (such as biotic stress, abiotic stress, and signalling molecules) and internal growth factors (Gustafson, 1946). Leaf senescence, an internal factor, is a common phenomenon during plant development (Lim et al., 2007). In agricultural production, some early-maturing cotton varieties tend to age prematurely, which severely affects their fibre yield and quality (Yu et al., 2005). We previously performed a genome-wide analysis of the WRKY gene family and found that WRKY TFs in cotton were differentially expressed during different stages of leaf senescence and under various stresses (Lin et al., 2015). These findings provide a basis for further exploring the involvement of WRKY TFs in leaf senescence- and stress-associated regulatory pathways in cotton.

The objective of our study was to examine the functional role of the GhWRKY91 TF in the regulation of leaf senescence and the drought stress response. Therefore, we isolated and characterized the *GhWRKY91* gene and analyzed its expression pattern. Our results showed that GhWRKY91 is involved in delayed natural and drought-induced leaf senescence, and increases drought tolerance in transgenic *Arabidopsis* plants. Furthermore, GhWRKY91 activates expression of its target gene *GhWRKY17*. Our findings reveal important functions of GhWRKY91 in leaf senescence and the response to drought stress, and provide a theoretical basis for developing cotton materials that present non-premature senescence and are stress tolerant.

## Materials and Methods

### Plant Materials and Growth Conditions

The cotton varieties CCRI10 (prematurely senescent), CCRI74 (prematurely senescent), and Liao4086 (non-prematurely senescent) were used in this study. Different tissues were sampled from the CCRI10 variety. To examine the functional role of *GhWRKY91* during leaf senescence, the expression profile of *GhWRKY91* was detected in 15-, 25-, 35-, 45-, 55-, and 65-day-old CCRI36 cotton leaves using transcriptome data (Lin et al., 2015). The data were normalized using the transcripts per million clean tags (TPM) algorithm (Lakhotia et al., 2014). The expression pattern of *GhWRKY91* was also measured in the top four leaves of the CCRI10 and Liao4086 varieties; the leaves were marked at the flowering stage and collected after one week at five notable development phases (7, 14, 21, 28, and 35 days). In addition, the expression patterns were measured in CCRI74 cotton leaves, which included newly flattened to nearly completely senescent leaves at five different development stages (stages 1–5) (Gu et al., 2018a).

With respect to ABA and drought treatments, CCRI10 seeds were germinated in soil in a greenhouse at 25 ± 1°C under a 16 h light/8 h dark photoperiod. Ten-day-old seedlings were treated with 200 μM ABA and 20% (w/v) polyethylene glycol 6000 (PEG6000) according to Gu et al. (2018b). The cotyledon samples were harvested at 0, 2, 4, 6, 8 and 12 h. Each sample contained three biological replicates.

The *Arabidopsis thaliana* Columbia ecotype (Col-0) was used as the wild type (WT). Transgenic plants expressing 35S::*GhWRKY91* or *ProGhWRKY91*::GUS were obtained using WT background plants. The seeds were surface sterilized and germinated on 1/2 Murashige and Skoog (1/2MS) (Murashige and Skoog, 1962) agar media in a growth chamber at 22°C under a 16 h light/8 h dark photoperiod. Two-week-old seedlings were then transplanted into soil in a greenhouse at 22 ± 1°C under a 16 h light/8 h dark photoperiod.

### Gene Cloning and Sequence Analysis

The full-length cDNA, genomic DNA, and promoter fragments of *GhWRKY91* were amplified from the cDNA and DNA products of CCRI10 cotton leaves. The PCR products were inserted into a clone vector, and the recombinant constructs were transferred into the DH5α strain for sequencing. The intron-exon structure was generated using GSDS 2.0 (http://gsds.cbi.pku.edu.cn/). The cis-elements were predicted using PlantCARE (http://bioinformatics.psb.ugent.be/webtools/plantcare/html). A multiple sequence alignment was carried out using DNAMAN software. Phylogenetic analyses were performed using the MEGA 7 program and the neighbour-joining method. All primers used in this study are listed in **Supplementary Table 1**.

### DNA, RNA Extraction, and Quantitative Real-Time PCR (qRT-PCR)

Genomic DNA was extracted *via* the cetyl-trimethylammonium bromide (CTAB) method as described previously (Porebski et al., 1997). Total RNA was isolated using an RNAprep Pure Plant Kit (DP441) (Tiangen, Beijing, China). The RNA was used as a template for cDNA synthesis *via* a PrimeScript™ RT Reagent Kit with gDNA Eraser (Perfect Real Time) (RR047A) (TaKaRa, Dalian, China). qRT-PCR was performed using SYBR^®^ Premix Ex Taq™ (Tli RNaseH Plus) (RR420A) (TaKaRa, Dalian, China) and an ABI 7500 Real-Time PCR System (Applied Biosystems, Foster City, CA, USA). The thermocycler programme consisted of pre-denaturation at 95°C for 30 s followed by 40 cycles at 95°C for 5 s and then 60°C for 34 s. Each sample was analyzed based on three technical replicates and the data were calculated in accordance with the 2^-ΔΔCt^ formula (Livak and Schmittgen, 2001). *Gossypium hirsutum Actin* (*GhActin*) and *Arabidopsis thaliana UBQ10* (*AtUBQ10*) were used as reference genes.

### Transcription Activation Assays

The *GhWRKY91* gene was cloned and inserted into the *Eco*RI and *Bam*HI sites of pGBKT7 to create pGBKT7-GhWRKY91 plasmids. The pGBKT7-GhWRKY91 and pGADT7 plasmids were co-transformed into Y2HGold cells. The transformed products were cultured and detected on SD-Trp-Leu (DDO), SD-Trp-Leu-His-Ade (QDO), and QDO/X-a-Gal (QDO/X) medium. The detailed protocol followed that of the Matchmaker™ Gold Yeast Two-Hybrid System (Clontech).

### Vector Construction and Genetic Transformation of *Arabidopsis thaliana*

The *GhWRKY91* gene was inserted into the *Bam*HI and *Sac*I sites of the binary vector pBI121 to generate 35S::*GhWRKY91* plasmids. To measure the promoter activity of *GhWRKY91*, a 2009 bp promoter fragment was inserted into the *Hin*dIII and *Bam*HI sites of the pBI121 vector to generate *ProGhWRKY91*::GUS plasmids. The 35S::*GhWRKY91* and *ProGhWRKY91*::GUS recombinant plasmids were subsequently introduced into *Agrobacterium tumefaciens* strain LBA4404. The LBA4404 cells harboring the fusion constructs were transformed into WT plants *via* the floral dip method (Clough and Bent, 1998). The positive plants were selected on 1/2MS medium containing kanamycin (100 mg/L), and further confirmed *via* PCR and qRT-PCR. T_3_ generation plants were used for phenotypic observation of leaf senescence and stress treatments.

### Analysis of Transgenic *Arabidopsis* Plants Under Normal, ABA, and Drought Conditions

To observe the phenotypes of transgenic plants under normal conditions, the seeds of the WT and three independent 35S::*GhWRKY91* lines (OE91-12, OE91-13, and OE91-20) were surface sterilized and germinated on 1/2MS agar medium. After two weeks, the seedlings were transplanted to soil, and the natural growth phenotype was observed. The rosette leaves from 50-day-old plants were sampled for qRT-PCR detection of senescence-associated genes (SAGs).

To investigate the ABA and drought tolerance of plants, each pot was divided into four sections on average. WT, OE91-12, OE91-13, and OE91-20 seedlings (8 seedlings per line) were planted in each section of the same pot to maintain the same growth conditions until the seedlings were three weeks old. Afterward, the seedlings were sprayed with 50 μM ABA and irrigated with 15% (w/v) PEG6000 (to mimic drought). Untreated seedlings were used as controls. Because of the different concentration sensitivities of different species to stress treatments, the treatment concentration of *Arabidopsis* was different from that of the cotton. The rosette leaves were collected and used to analyze the expression of SAGs, and ABA- and stress-related genes. In addition, the plants were subjected to a water shortage treatment. Irrigation was withheld for four-week-old WT, OE91-12, OE91-13, and OE91-20 plants for approximately 2 weeks. The plants were imaged after their main stems were cut and removed. Three replicates were included per treatment.

### β-Glucuronidase (GUS) Histochemical Staining

To investigate the promoter activity in different tissues, 8-day-old seedlings, stems, leaves, flower buds, flowers, and fruit pods from *ProGhWRKY91*::GUS plants were used for GUS staining. For the stress treatments, two-week-old *ProGhWRKY91*::GUS seedlings were treated in 1/2MS liquid medium that was supplemented with or without 100 μM ABA and 200 mM mannitol. Due to the difference in experiment purposes, the stress concentration was different from that of overexpression lines. GUS staining was performed as described previously (Jefferson et al., 1987). The treated samples were immersed in GUS histochemical staining buffers [0.1 mM NaPO_4_ (pH 7.0), 10 mM EDTA-Na_2_ (pH 8.0), 0.1% Triton X-100, 1 mM K_3_Fe(CN)_6_, and 2 mM X-Gluc], and subsequently incubated at 37°C overnight. After staining, the samples were decolorized in 75% ethanol until the color of the negative control turned white. GUS activity was estimated based on the presence of blue. In addition, the GUS staining assay is representative of the results in two independent transgenic lines.

### Yeast One Hybrid (Y1H) Assays

The *GhWRKY91* gene was cloned into pGADT7 vector at the *Eco*RI and *Bam*HI sites to create pGADT7-GhWRKY91 prey plasmids. Three copies of specific fragments from the promoter regions of *GhWRKY3*, *GhWRKY17*, *GhWRKY25*, *GhWRKY27a*, *GhWRKY68*, *ASCORBATE PEROXIDASE 1* (*GhAPX1*), and *RESPONSIVE TO DESICCATION 22* (*GhRD22*) were cloned into a pHIS2 vector to generate bait carriers (**Supplementary Table 2**). The pGADT7-GhWRKY91 construct and each bait carrier were subsequently co-transformed into Y187 yeast cells. The transformed yeast cells were grown and detected on DDO and SD-Trp-Leu-His (TDO) medium that were supplemented with 200 mM 3-amino-1,2,4-triazole (3-AT) (TDO + 200 mM 3-AT) to evaluate protein-DNA interactions based on growth ability.

### Electrophoretic Mobility Shift Assays (EMSAs)

The *GhWRKY91* gene was cloned into pGEX-4T-1 to produce pGEX-4T-1-GhWRKY91 constructs, which were fused to a glutathione S-transferase (GST) tag at the N-terminus. The pGEX-4T-1-GhWRKY91 constructs were transformed into the *E. coli* strain Arctic-Express™, after which, the cells containing the fusion plasmids were induced by 0.5 mM isopropyl-β-D-thiogalactoside (IPTG) at 20°C for 4 h at 220 r/min. The fusion proteins were purified with a GST Fusion Protein Purification Kit (GenScript) and digested to remove any GST tags. Any biotin end-labelled probes containing a W-box originated from the promoter regions of *GhWRKY17*, *GhWRKY27a*, *GhWRKY68*, and *GhAPX1*. Unlabelled probes were used as cold competitors. We performed the EMSAs using a LightShift^®^ Chemiluminescent EMSA Kit (Thermo Scientific, Waltham, MA, USA).

### Dual-Luciferase Reporter Assay

The *GhWRKY91* gene was cloned into the pGreenII 62-SK effecter vector, and the *GhWRKY17* promoter was cloned into pGreenII0800-LUC reporter vector. The recombinant plasmids were transformed into the GV3101 (pSoup-p19) strain. The culture was incubated to an OD600 value of 0.8 and adjusted to an OD600 value of 0.6 with the infiltration buffer (10 mM MgCl_2_, 10 mM MES, and 100 μM acetosyringone). After resting at room temperature for 3 h, the suspensions of effecter and reporter were mixed in a 9:1 ratio and co-infiltrated into tobacco leaves according to the method by Hellens et al. (Hellens et al., 2005). Three days later, the LUC and REN values were obtained using a Dual-Luciferase^®^ Reporter Assay System (Promega, USA) on a GloMax 20/20 Luminometer (Promega, USA). The trans-activation of the GhWRKY91 TF to *GhWRKY17* promoter was indicated by the LUC/REN ratio. At least six independent repeats were performed.

## Results

### Cloning and Sequence Analysis of GhWRKY91

**Figure 1:**
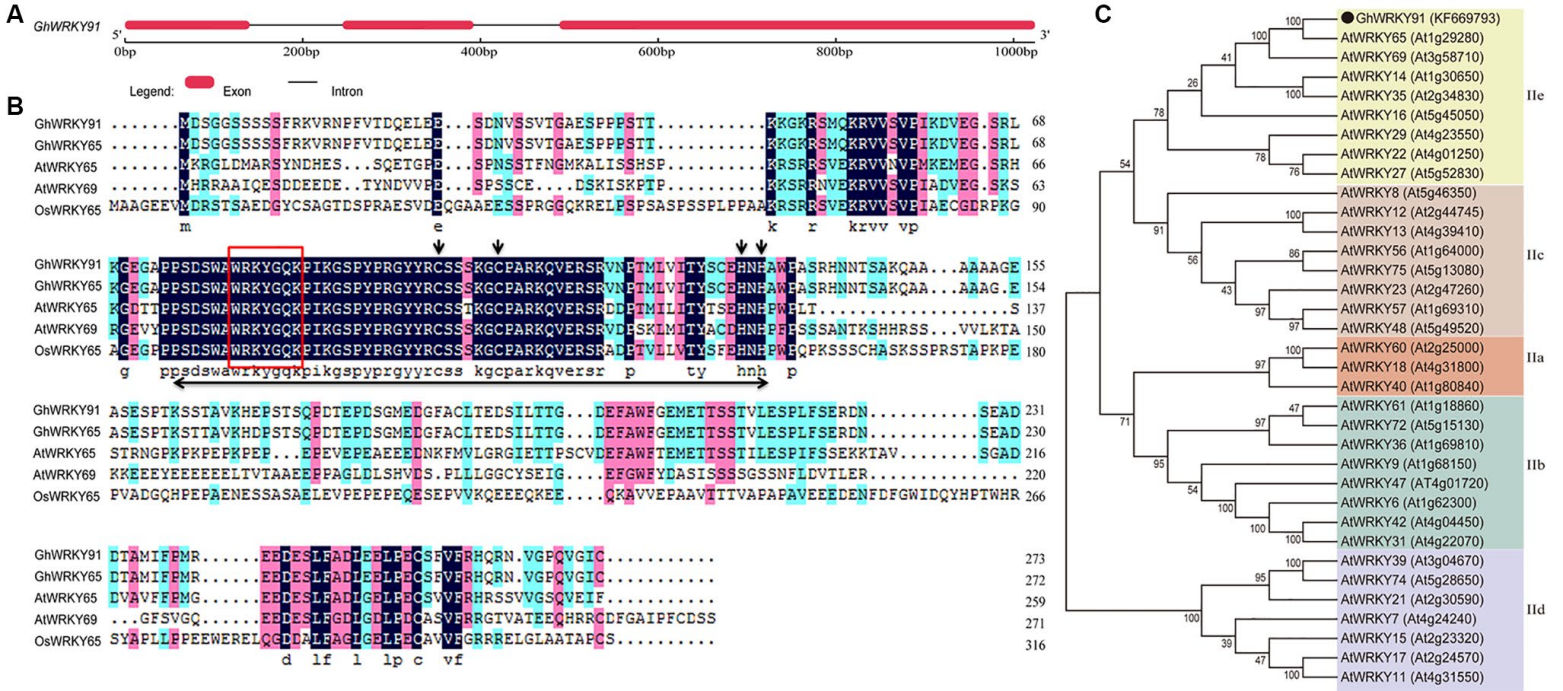
Sequence and phylogenetic analyses of GhWRKY91. **(A)** Exon-intron structure of the *GhWRKY91* gene. The thick red line represents an exon, and the black line represents an intron. **(B)** Sequence alignment of the GhWRKY91 protein with the closely related proteins GhWRKY65 (Gh_D10G0329.1), AtWRKY65 (AT1G29280.1), AtWRKY69 (AT3G58710.1), and OsWRKY65 (XP_015647591.1). The WRKY domain is indicated by a double-headed arrow. The highly conserved core sequence WRKYGQK in the WRKY domain is represented by a red box. The C and H residues in the zinc finger motif are indicated by downward arrows. **(C)** Phylogenetic relationship of the GhWRKY91 protein with other group II WRKY proteins from *Arabidopsis*. GhWRKY91 is indicated by the black dot. The abbreviations before the gene names are as follows: Gh, *Gossypium hirsutum*; At, *Arabidopsis thaliana*; and Os, *Oryza sativa*.

The genomic DNA and full-length cDNA sequences of *GhWRKY91* (GenBank accession number: KF669793) were amplified from the cotton variety CCRI10. The *GhWRKY91* gene contained three exons and two introns, and the genomic DNA sequence was interrupted by two introns of 105 and 97 bp at the ‘GG’ and ‘GT’ sites (**Figure 1A**). The open reading frame of *GhWRKY91* was 822 bp in length and encoded 273 amino acid residues. The estimated molecular mass of the GhWRKY91 protein was 29.82 kDa, and the isoelectric point was 5.13. A comparison of the protein sequences of GhWRKY91 with its related proteins from different species demonstrated that GhWRKY91 was 98.53% homologous to GhWRKY65 (Gh_D10G0329.1), 50.71% homologous to AtWRKY65 (AT1G29280.1), 39.16% homologous to AtWRKY69 (AT3G58710.1), and 33.54% homologous to OsWRKY65 (XP_015647591.1). A multiple sequence alignment revealed that the GhWRKY91 protein includes one WRKY domain that comprises approximately 60 amino acids with a highly conserved amino acid sequence (WRKYGQK) and a C2H2 putative zinc finger motif, indicating that GhWRKY91 belongs to the group II family (**Figure 1B**). According to the phylogenetic tree, group II members could be divided into five subgroups (IIa-IIe), and GhWRKY91 was closely related to group IIe members (**Figure 1C**).

### Transcriptional Activity of GhWRKY91

**Figure 2:**
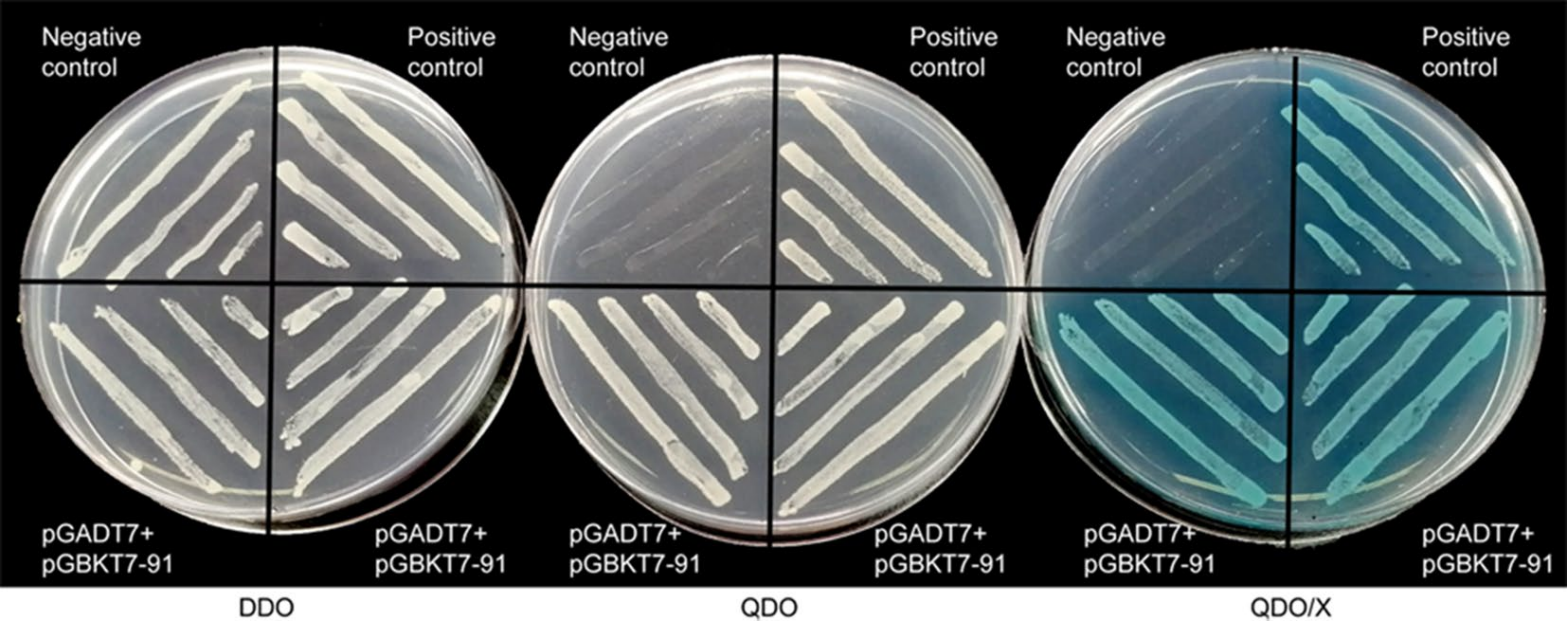
Transcriptional activity of GhWRKY91 in Y2HGold yeast cells. Yeast cells were assayed on DDO (SD-Trp-Leu), QDO (SD-Trp-Leu-His-Ade) and QDO/X (SD-Trp-Leu-His-Ade/X-a-Gal) medium. The combination of the pGADT7-large T and pGBKT7-p53 plasmids was used as a positive control. The combination of the pGADT7-large T and pGBKT7-laminC plasmids was used as a negative control.

A transcriptional activation assay was performed *in vitro* to demonstrate whether GhWRKY91 has transcription activation activity in yeast cells. The plasmids of the experimental group (pGADT7+pGBKT7-GhWRKY91), positive group, and negative group were transformed into Y2HGold yeast cells and assayed on DDO, QDO, and QDO/X agar medium. The results showed that all transformation products can grow normally on DDO medium (**Figure 2**). The experimental group and positive group grew well on QDO medium and turned blue on QDO/X medium, whereas the negative group could not; indicating that GhWRKY91 could autonomously activate the reporter genes in the absence of a prey protein (**Figure 2**).

### Expression Patterns of *GhWRKY91* During Leaf Senescence and Under Stress Treatments

**Figure 3:**
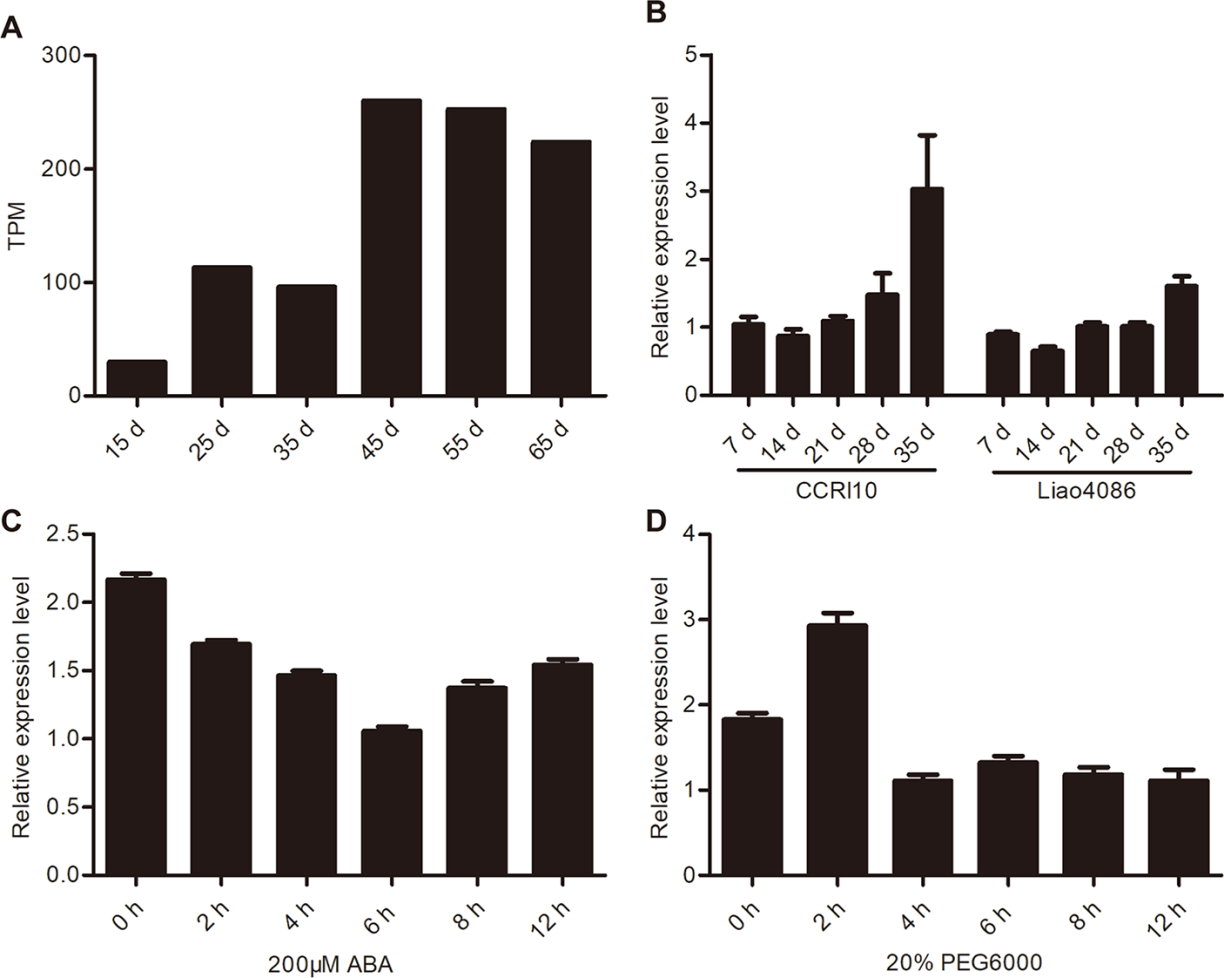
Expression patterns of *GhWRKY91* during leaf senescence and under stress treatments. **(A)** Expression profiles of *GhWRKY91* in 15-, 25-, 35-, 45-, 55-, and 65-day-old CCRI36 cotton leaves using the transcriptome data (Lin et al., 2015). TPM, transcripts per million clean tags. **(B)** Transcript levels of *GhWRKY91* at different stages of leaf senescence in CCRI10 and Liao4086 varieties. **(C–D)** Transcript levels of *GhWRKY91* under ABA and PEG6000 treatments. *GhActin* served as the reference gene. The data are the means ± standard errors (SEs) of three biological replicates.

The transcriptome data (Lin et al., 2015) showed that *GhWRKY91* transcripts increased as the CCRI36 cotton leaves aged and was highly expressed in senescent leaves (**Figure 3A**). In addition, the expression of *GhWRKY91* increased gradually with leaf senescence and was more prevalent in the premature-senescence cotton variety CCRI10 than in the non-premature-senescence variety Liao4086 (**Figure 3B**). With respect to ABA stress, ABA treatment repressed the expression of *GhWRKY91* in a time series of 2–12 h (**Figure 3C**). With respect to the drought treatment, the *GhWRKY91* transcripts accumulated immediately and peaked at 2 h after the initial treatment, followed by a decrease in accumulation throughout the remaining 4–12 h (**Figure 3D**).

### *GhWRKY91* Promoter Analysis

The cis-elements in the *GhWRKY91* promoter were predicted using PlantCARE. The results revealed that stress response-, light response-, and development-related elements were present in this region (**Supplementary Table 3**). The stress responsive elements included ABA (ABRE, ACGTG), low temperature (LTR, CCGAAA), and anaerobic (ARE, AAACCA) associated elements (**Supplementary Table 3**).

In eight-day-old seedlings, GUS staining was observed mainly in the cotyledons, hypocotyls, and upper parts of the roots (**Supplementary Figure 1A**). At the vegetative stage, GUS staining was found in the stems and leaves, especially at the edge of the leaves (**Supplementary Figure 1B, C**). However, during the generative growth phase, GUS activity was almost undetectable in the flower, buds, and fruit pods (**Supplementary Figure 1D–F**).

**Figure 4:**
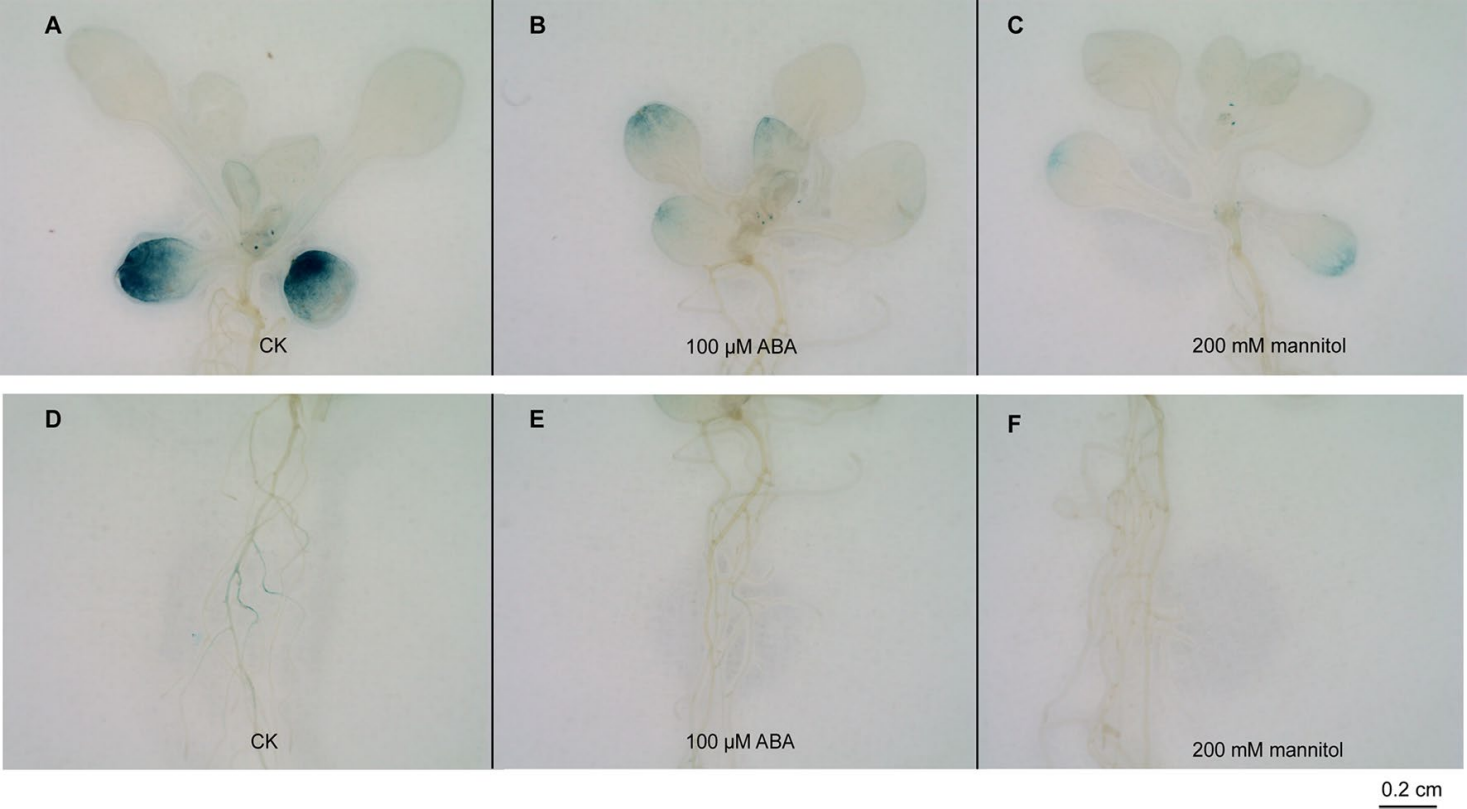
Histochemical GUS assays in *ProGhWRKY91*::GUS transgenic *Arabidopsis* plants. **(A–C)** GUS staining of the leaves under control, ABA, and mannitol treatments. **(D–F)** GUS staining of the roots under control, ABA, and mannitol treatments. Bar = 0.2 cm.

GUS activity was strongly expressed in cotyledons but weakly expressed in rosette leaves in the control (**Figure 4A**). After the ABA and mannitol treatments, the GUS signal was weak in the cotyledons and rosette leaves compared with the control seedlings (**Figures 4B, C**). Similarly, GUS staining was inconspicuous in the roots treated with ABA and drought compared with the control (**Figures 4D–F**).

### Overexpression of *GhWRKY91* Delayed Leaf Senescence in Transgenic *Arabidopsis* Plants

**Figure 5:**
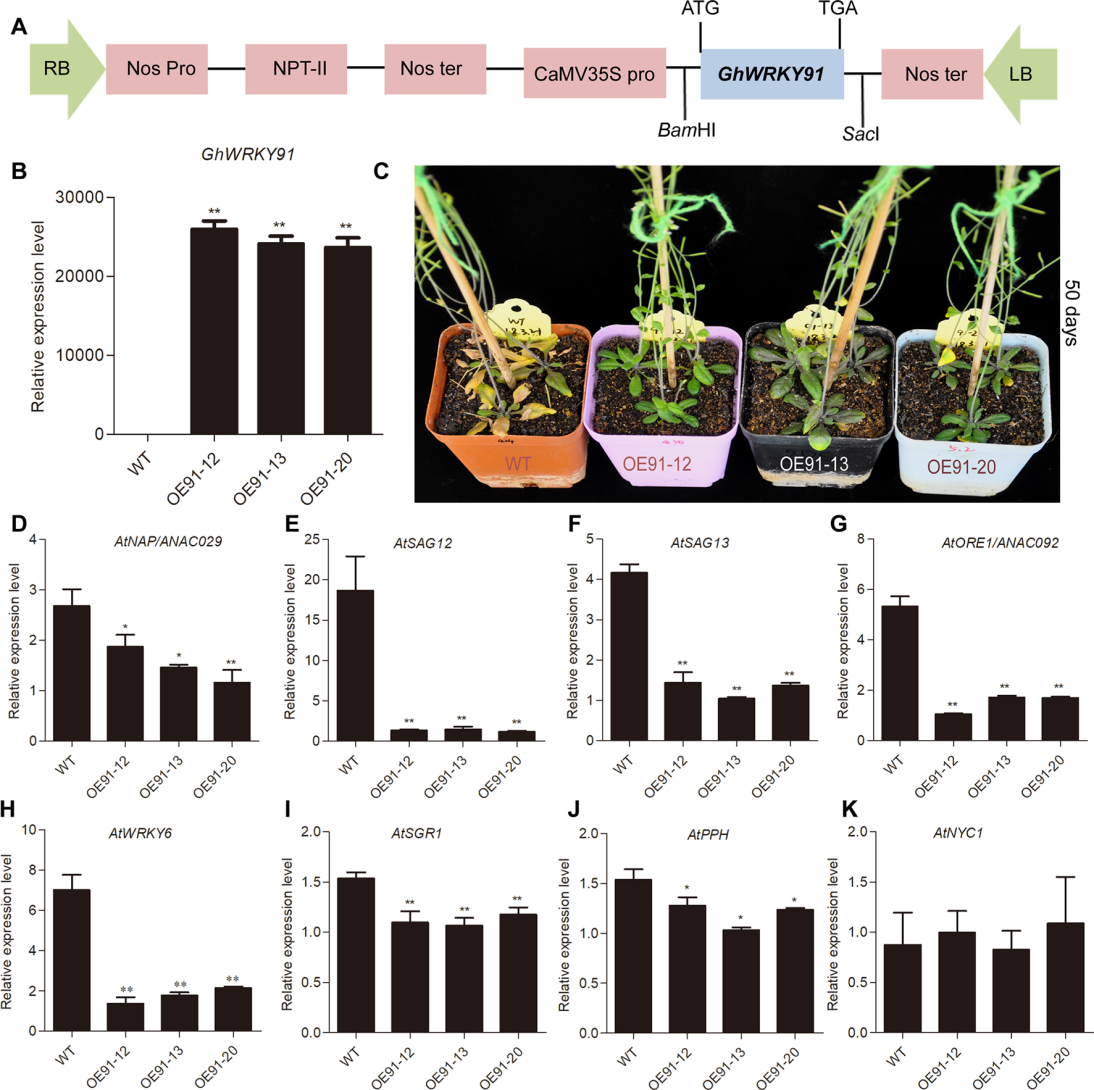
Overexpression of *GhWRKY91* in *Arabidopsis* plants delayed leaf senescence. **(A)** The T-DNA construction diagram used for *Arabidopsis* transformation. **(B)** Transcript levels of *GhWRKY91* in WT and transgenic plants. **(C)** Phenotypic characteristics of WT and transgenic plants grown for 50 days. The seeds of WT and transgenic lines were germinated on 1/2MS agar media in a growth chamber at 22°C under a 16 h light/8 h dark photoperiod. Two-week-old seedlings were then transplanted into soil in a greenhouse at 22 ± 1°C under a 16 h light/8 h dark photoperiod, and the natural senescence phenotype was observed in 50-day-old plants. **(D**–**K)** Transcript levels of SAGs in the rosette leaves of WT and transgenic plants grown for 50 days. *AtUBQ10* served as the reference gene. The data are the means ± SEs of three biological replicates. **P < 0.01 and *P < 0.05.

To evaluate the influence of the *GhWRKY91* gene on plants, we overexpressed *GhWRKY91* in *Arabidopsis* plants. The transgenic lines (OE91-12, OE91-13 and OE91-20) containing T-DNA with 35S::*GhWRKY91* were generated (**Figure 5A**) and confirmed by PCR (**Supplementary Figure 2A**) and qRT-PCR (**Figure 5B**). The 21-day-old transgenic plants were relatively smaller in size than the WT plants (**Supplementary Figure 2B**). The 35-day-old transgenic *Arabidopsis* plants exhibited delayed flowering and leaf senescence compared with the WT plants (**Supplementary Figure 2B**). When the plants were 50 days old, the WT plants became severely senescent and yellow, while the transgenic plants were still green with only a small amount of yellowing (**Figure 5C**). In addition, we assessed the transcript levels of SAGs that are up-regulated factors during leaf senescence in the 50-day-old plants. The results showed that the transcript levels of SAGs *AtNAP/ANAC029* (AT1G69490), *AtSAG12* (AT5G45890), *AtSAG13* (AT2G29350), *AtORE1/ANAC092* (AT5G39610), *AtWRKY6* (AT1G62300), *STAY-GREEN1* (*AtSGR1*) (AT4G22920), and *PHEOPHYTIN PHEOPHORBIDE HYDROLASE* (*AtPPH*) (AT5G13800) were significantly lower in the transgenic plants than in the WT plants (**Figure 5D–J**). However, there was no difference in the transcript levels of *NON-YELLOW COLORING 1* (*AtNYC1*) (AT4G13250) between the WT and transgenic plants (**Figure 5K**).

### Overexpression of *GhWRKY91* Improved Drought Tolerance in Transgenic *Arabidopsis* Plants

**Figure 6:**
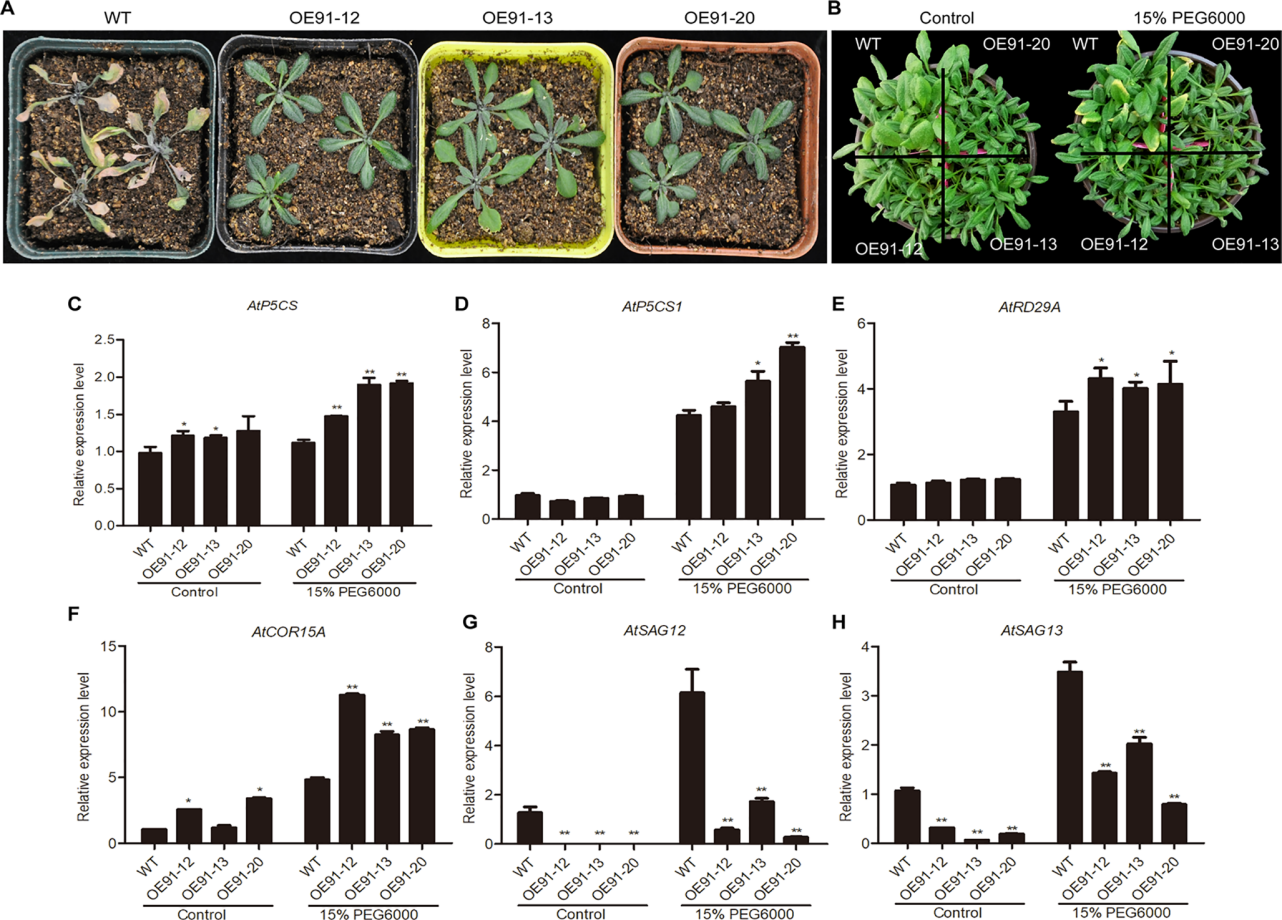
Overexpression of *GhWRKY91* in *Arabidopsis* plants improved drought tolerance. **(A)** Phenotypic characteristics of six-week-old WT and transgenic plants under water deficit conditions. Four-week-old WT and transgenic plants grown in soil were subjected to a water shortage treatment for approximately two weeks. **(B)** Phenotypic characteristics of four-week-old WT and transgenic plants under the control and PEG6000 treatments. Three-week-old WT and transgenic plants grown in soil were irrigated with 15% PEG6000 for one week. Plants grown under normal growth conditions severed as controls. All the plants were grown in a greenhouse at 22 ± 1°C under a 16 h light/8 h dark approximately. **(C–F)** Expression levels of the stress-related genes *AtP5CS*, *AtP5CS1*, *AtRD29A*, and *AtCOR15A* in four-week-old WT and transgenic plants. **(G–H)** Expression levels of the SAGs *AtSAG12* and *AtSAG13* in the WT and transgenic plants. *AtUBQ10* was used as the reference control. The data are the means ± SEs of three biological replicates. ** P < 0.01 and * P < 0.05.

Four-week-old plants in pots were used for the water deficit treatment. After water was withheld for two weeks, the WT plants showed definitive wilting and yellowing, while the transgenic plants were still very green (**Figure 6A**). Three-week-old plants were subjected to a 15% PEG6000 treatment, and after one week, the WT plants displayed a greater extent of yellow leaves than the transgenic plants (**Figure 6B**).

The expression levels of stress-inducible genes (*DELTA-1-PYRROLINE-5-CARBOXYLATE SYNTHETASE* (*AtP5CS*), AT2G39800; *AtP5CS1*, AT2G39800; *AtRD29A*, AT5G52310; and *COLD-REGULATED 15A* (*AtCOR15A*), AT2G42540) and SAGs (*AtSAG12*; *AtSAG13*) were examined in the presence and absence of 15% PEG6000. Generally, the expression levels of *AtP5CS*, *AtP5CS1*, *AtRD29A*, and *AtCOR15A* were similar or higher in the transgenic plants than in the WT plants under normal conditions but were significantly elevated in the transgenic plants compared with the WT plants in response to the PEG6000 treatment (**Figures 6C–F**). The expression levels of *AtSAG12* and *AtSAG13* were markedly lower in the transgenic plants than the WT plants under normal conditions. However, although the expression levels of the two genes were elevated in the presence of 15% PEG6000, they were still significantly lower in the transgenic plants than in the WT plants (**Figures 6G, H**).

### GhWRKY91 Bound Directly to the Promoter of *GhWRKY17 and* Trans-Activated *GhWRKY17* Expression

**Figure 7:**
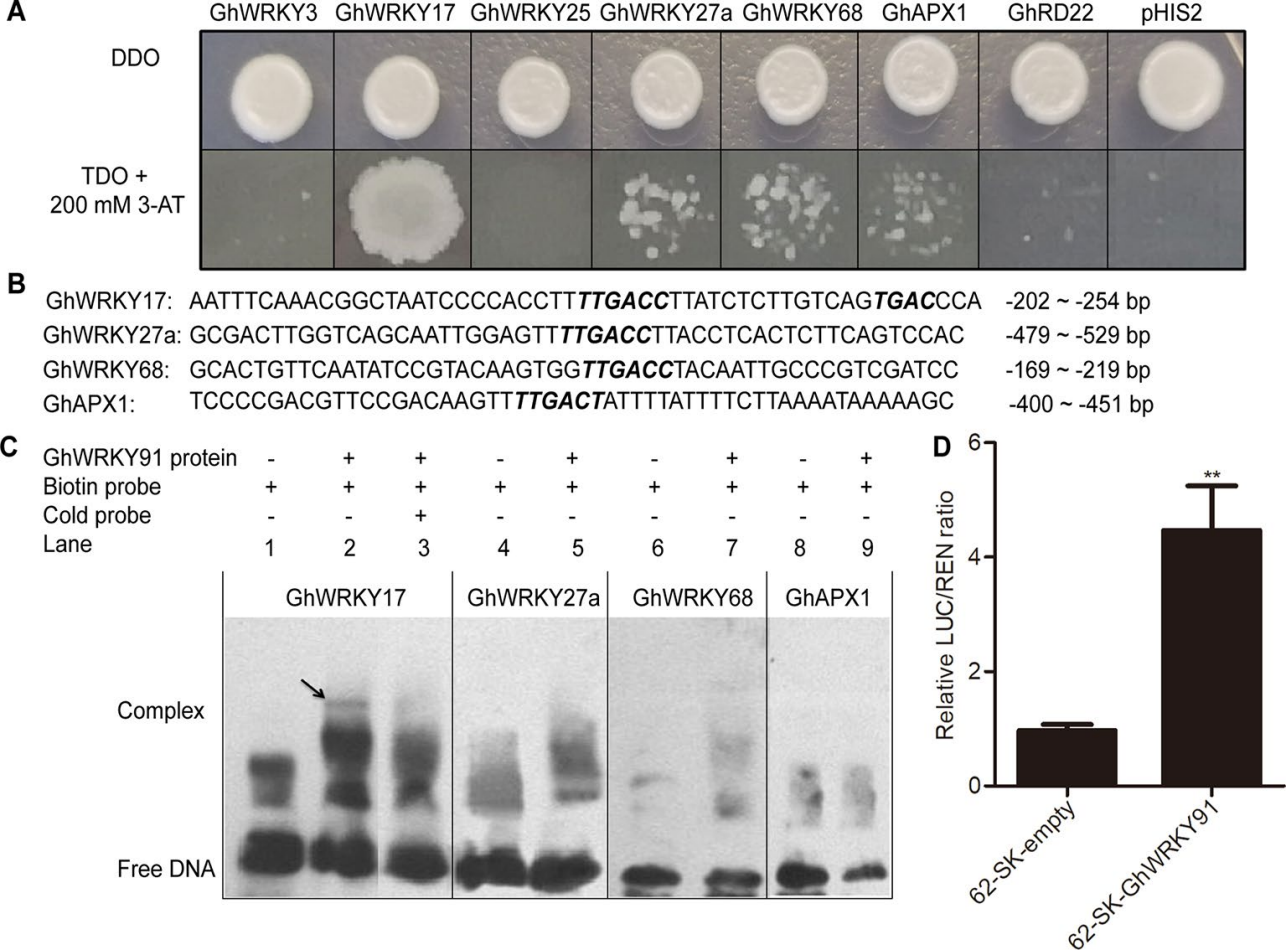
GhWRKY91 directly targets the promoter of *GhWRKY17* and trans-activates *GhWRKY17* expression. **(A)** Interaction between GhWRKY91 and the promoters of candidate target genes *GhWRKY3*, *GhWRKY17*, *GhWRKY25*, *GhWRKY27a*, *GhWRKY68*, *GhAPX1*, and *GhRD22* in Y1H system. The combination of pGADT7-GhWRKY91 and pHIS2 plasmids was used as a negative control. **(B)** Probe sequences of *GhWRKY17*, *GhWRKY27a*, *GhWRKY68*, and *GhAPX1* for the EMSA assays. **(C)** Binding of GhWRKY91 to the W-boxes in the promoters of *GhWRKY17*, *GhWRKY27a*, *GhWRKY68*, and *GhAPX1* in EMSA assays. ‘–’ indicates absence, while ‘+’ indicates presence. **(D)** GhWRKY91 trans-activates *GhWRKY17* promoter in transient dual-luciferase reporter system. The LUC/REN ratio of the combination of pGreenII-62-SK empty vector and *GhWRKY17* promoter was set as 1. The data are the means ± SEs of six biological replicates. **P < 0.01.

Previous studies showed that *GhWRKY3* (Guo et al., 2011), *GhWRKY17* (Yan et al., 2014), *GhWRKY25* (Liu et al., 2016), *GhWRKY27a* (Yan et al., 2015), and *GhWRKY68* (Jia et al., 2015) were involved in the regulation of salt, drought, and ABA responses through mediating ROS production and ABA signalling. The *APX* gene encodes an ROS-scavenging enzyme that catalyses the reduction of hydrogen peroxide (Ozyigit et al., 2016), and RD22 is involved in drought tolerance (Harshavardhan et al., 2014). To identify the regulatory mechanism of GhWRKY91, we predicted the W-boxes in the promoters of these genes and found that W-boxes were present in their promoters, indicating the potential roles of these genes as targets. In the Y1H system, the transformation product containing GhWRKY91 and the special fragment in the promoter region of *GhWRKY17* grew well on TDO + 200 mM 3-AT selective medium, whereas the others did not grow or only displayed defective spots (**Figure 7A**).

For the EMSA assays, the probes and sequence locations in the region upstream of the ATG initiation site of the genes (*GhWRKY17*, *GhWRKY27a*, *GhWRKY68*, and *GhAPX1*) are presented in **Figure 7B**. The lanes containing only biotin-labelled probes were used as negative controls (**Figure 7C**; lanes 1, 4, 6, and 8). None of the probes from the *GhWRKY27a*, *GhWRKY68*, or *GhAPX1* genes were bound by the GhWRKY91 protein to produce delayed bands (**Figure 7C**; lanes 5, 7, 9). However, the labelled probes of *GhWRKY17* could be recognized and bound by the GhWRKY91 protein, thus causing a mobility shift (**Figure 7C**; lane 2). The unlabelled probes of *GhWRKY17* were overdosed as cold competitors; in these cases, most of the labelled wild probes bound by the GhWRKY91 protein were competitively replaced by unlabelled wild probes, resulting in the disappearance of the bands (**Figure 7C**; lane 3), indicating that GhWRKY91 could bind to the promoter of *GhWRKY17*.

To elucidate the relationship of GhWRKY91 in the regulation of *GhWRKY17* expression, a transient dual-luciferase assay was conducted in tobacco. The control data (pGreenII 62-SK empty vector pus the *GhWRKY17* promoter) were set to 1, and the experimental group data were the ratio to the control group. As a result, as shown in **Figure 7D**, the LUC/REN ratio was extremely significantly increased in the experimental group and 4.61 times that of the control group.

## Discussion

Growing evidence has shown that WRKY TFs are widely involved in plant development, leaf senescence and various abiotic/biotic stress responses (Lim et al., 2007; Yu et al., 2012; Xiao et al., 2013; Pan and Jiang, 2014). However, the function roles of WRKY TFs remain to be explored in cotton plants. In the present study, we isolated the WRKY TF GhWRKY91 from cotton and characterized its functional roles. A sequence analysis revealed that the GhWRKY91 protein has one WRKY domain with a C2H2 zinc finger motif, indicating that GhWRKY91 is a group II member according to the criteria of Eulgem et al. (2000). A phylogenetic analysis revealed that GhWRKY91 clustered with group IIe WRKY TFs from *Arabidopsis*, which were further classified into the group IIe subfamily.

Some of WRKY TFs were shown to be important senescence regulators in plants (Lim et al., 2007; Besseau et al., 2012). To characterize the functional role of GhWRKY91 in leaf senescence, we examined the transcripts of *GhWRKY91* at different stages of leaf senescence in cotton. We observed that the expression of *GhWRKY91* increased as leaf senescence progressed and was highly expressed in senescent leaves, implying that *GhWRKY91* may serve as a senescence-related gene in cotton. In addition, the expression level of *GhWRKY91* was higher in the premature-senescence variety CCRI10 than in the non-premature-senescence variety Liao4086. Previous studies have shown that *GhNAC79* (Guo et al., 2017b) and *GhNAC12* (Zhao et al., 2016) are highly expressed in premature-senescence varieties, and that *Arabidopsis* plants transformed with these genes positively regulate age-triggered leaf senescence, which prompted us to explore the relationship between GhWRKY91 and leaf senescence in transgenic materials further.

Previous studies shown that the senescence characteristics of *Arabidopsis* plants can be further verified by the expression of SAGs (Kim et al., 2013; Chen et al., 2017). In our study, the senescence-positive SAGs including *AtNAP/ANAC029* (Guo and Gan, 2006), *AtWRKY6* (Robatzek and Somssich, 2002), and *AtORE1/ANAC092* (Kim et al., 2009) and the chlorophyll degradation-related genes *AtSGR1* (Sakuraba et al., 2014b) and *AtPPH* (Schelbert et al., 2009), *AtSAG12* (James et al., 2018), and *AtSAG13* (Chen et al., 2017) were investigated. Transgenic *Arabidopsis* plants overexpressing *GhWRKY91* display a delayed-leaf senescence phenotype that corresponded with the reduced expression of these SAGs, suggesting that *GhWRKY91* might negatively regulate leaf senescence. In addition, among these genes, AtWRKY6 interacts with the gibberellin (GA) signalling component/DELLA protein RGA to repress the transcriptional activation of AtWRKY6 on downstream SAGs in dark-induced leaf senescence in *Arabidopsis* (Robatzek and Somssich, 2002; Zhang et al., 2018). *AtORE1/ANAC092* is regulated by ethylene signalling (Kim et al., 2009), ABA signalling (Sakuraba et al., 2014a), and the circadian rhythm (Kim et al., 2018), and it directly regulates a number of genes related to SAGs (Woo et al., 2019). These studies suggest that *GhWRKY91* might affect hormone-related signalling pathways during leaf senescence. Moreover, differential SAG expression detected in the different transgenic lines may be due to independent transformation events and positional effects of the T-DNA insertions (Negi et al., 2015). *AtNYC1* encodes a chlorophyll b reductase that is involved in the degradation of chlorophyll b and LHCII (light harvesting complex II) (Horie et al., 2009). However, the expression of *AtNYC1* did not differ between WT and transgenic lines, indicating the complexity of the regulatory mechanism of leaf senescence. In addition, some studies have linked ABA and drought to plant leaf senescence (Woo et al., 2019). For example, the SNAC-A (A subfamily of stress-responsive NAC) septuple (*anac055anac019anac072anac002anac081anac102anac032*) mutant exhibits delayed ABA-induced leaf senescence in *Arabidopsis* (Takasaki et al., 2015). In barley, WHIRLY1 knockdown lines exhibit delayed drought-induced leaf senescence (Janack et al., 2016). However, leaf senescence associated with WRKY TFs under ABA and drought stress conditions is largely unknown in cotton. Here, *GhWRKY91* was found to delay ABA- and drought-induced leaf senescence in transgenic *Arabidopsis* plants (**Supplementary Figure 3** and **Figure 6**). Taken together, our results suggest that GhWRKY91 might serve as a negative regulator during natural leaf senescence and during ABA- and drought-induced leaf senescence, thus expanding the functional roles of WRKY TFs during leaf senescence in cotton. Our results also showed that the maximum expression of *GhWRKY91* occurred in the roots and not in the leaves, indicating that this gene may have other functions yet to be discovered.

The expression of *GhWRKY91* was downregulated by ABA and drought treatment in cotton and verified by GUS activity in transgenic *Arabidopsis* plants containing the *GhWRKY91* promoter, indicating that *GhWRKY91* expression might be suppressed by ABA and drought. To further study the relationship of ABA and drought stress with *GhWRKY91*, *GhWRKY91-*overexpressing plants were subjected to ABA and drought treatments and the transgenic plants exhibited delayed ABA-induced leaf senescence and improved drought tolerance. To gain further insight into the mechanism of *GhWRKY91* in the ABA response, the transcripts of positive senescence regulators (*AtWRKY53*, *AtSAG13*, and *AtPPH*) and ABA-responsive genes (*HYPERSENSITIVE TO ABA1* (*AtHAB1*), *ABA INSENSITIVE 1* (*AtABI1*), *ABA-RESPONSIVE ELEMENT BINDING PROTEIN 1* (*AtAREB1/ABF2)*, and *AtAREB2/ABF4*) were investigated. After ABA treatment, the expression levels of *AtWRKY53*, *AtSAG13*, and *AtPPH* were significantly lower in transgenic plants than in WT (**Supplementary Figure 3B–D**), indicating that *GhWRKY91* could delay ABA-induced leaf senescence. Exogenous ABA application can alter the expression of ABA-responsive genes (Himmelbach et al., 2003). *AtHAB1* and *AtABI1* are members of the protein phosphatase 2C (PP2C) family and involved in the negative regulation of ABA signalling (Merlot et al., 2001; Saez et al., 2004). AREB/ABFs, which are positive regulators in the ABA signalling pathway, bind to ABA-responsive elements in the promoter of ABA-inducible genes (Leite et al., 2014). The transcript levels of negative regulators *AtHAB1* and *AtABI1* were significantly higher in transgenic plants than WT (**Supplementary Figures 3E, F**). However, the expression levels of positive regulators *AtAREB1/ABF2* and *AtAREB2/ABF4* were also higher in transgenic plants than in WT (**Supplementary Figure 3G–H**), suggesting a contradictory mechanism in delayed leaf senescence. In our study, overexpression of *GhWRKY91* in *Arabidopsis* caused marginal stunting (**Supplementary Figure 2B**). In our study, the transcript levels of *AtAREB1*/*ABF2* and *AtAREB2*/*ABF4* were upregulated in transgenic plants. Previous studies showed that the overexpression of the *Arabidopsis AtAREB1*/*ABF2* or *AtAREB2*/*ABF4* genes in potato plants caused short and stunted growth (Garcia et al., 2014). Therefore, high expression of *AtAREB1/ABF2* and *AtAREB2/ABF4* may be associated with stunting in transgenic plants, thus leading to delayed leaf senescence. Moreover, the ABA responsive *cis*-element ABRE (ACGTG) was presented in the promoter region of *GhWRKY91*, suggesting the probable role of *GhWRKY91* in plant development by ABA signalling.

To explore the mechanism of *GhWRKY91* in drought tolerance, the stress-related genes *AtP5CS*, *AtP5CS1*, *AtRD29A*, and *AtCOR15A* were identified. Plants invoke various resistance mechanisms to manage various environmental stresses (Ashraf and Foolad, 2007; Shi et al., 2014). P5CS is a key enzyme involved in the synthesis of proline (Hu et al., 1992; Ashraf and Foolad, 2007), which can stabilize metabolic processes in protoplast colloids and prevent dehydration of cells (Hu et al., 1992; Ashraf and Foolad, 2007). Increased enzymatic activity of P5CS leads to increased biosynthesis of proline, thus improving plant resistance to stress (Yamchi et al., 2007). *RD29A*, which encodes a hydrophilic protein, is strongly induced by ABA, drought, and salt stress (Msanne et al., 2011). In addition, DRE and ABRE motifs are present in the promoter of *RD29A*, suggesting important roles for *RD29A* during drought and the ABA response (Yamaguchi-Shinozaki and Shinozaki, 1993). The *COR15A* gene is considered a marker for drought stress and participates in stress responses *via* an ABA-dependent signalling pathway (Meng et al., 2015). In our study, *GhWRKY91-*overexpressing plants exhibited improved drought tolerance, and the expression levels of stress-related genes were significantly higher in the transgenic plants, indicating that *GhWRKY91* may positively regulate drought tolerance *via* ABA signalling pathways and some small molecules.

WRKY TFs can specifically bind to W-box [TTGAC(C/T)] elements in the promoters of target genes to activate or inhibit their expression (Rushton et al., 2010). WRKY genes usually contain W-box cis-elements that can combine with other WRKY TFs or the gene itself (Skibbe et al., 2008; van Verk et al., 2011). Previous reports showed that ectopic overexpression of *GhWRKY17* increases transgenic tobacco plant sensitivity to ABA, drought, and salt stress by participating in the ABA signalling pathway and affecting the antioxidant enzyme system (Yan et al., 2014). Our results showed that W-box elements are indeed present in the *GhWRKY17* promoter. In addition, *GhWRKY17* was targeted directly by GhWRKY91, suggesting that GhWRKY91 may regulate the transcriptional expression of *GhWRKY17*. The dual-luciferase reporter assay demonstrated that GhWRKY91 activates the expression of *GhWRKY17*. Thus, GhWRKY91 may be involved in the ABA regulatory network and may mediate the intracellular ROS balance *via* transcriptional activation of *GhWRKY17* expression. Expression analysis revealed that the *GhWRKY17* transcripts were downregulated by leaf senescence (**Supplementary Figure 4**), but induced in response to ABA and drought treatments (Yan et al., 2014). The expression of *GhWRKY91* was upregulated by leaf senescence and downregulated by ABA and drought. We found that *GhWRKY91* and *GhWRKY17* presented opposite expression trends under leaf senescence, ABA and drought stress. Therefore, we speculated that there may be some genes that inhibit *GhWRKY17* expression activated by GhWRKY91. However, additional experiments are needed to validate this hypothesis.

**Figure 8:**
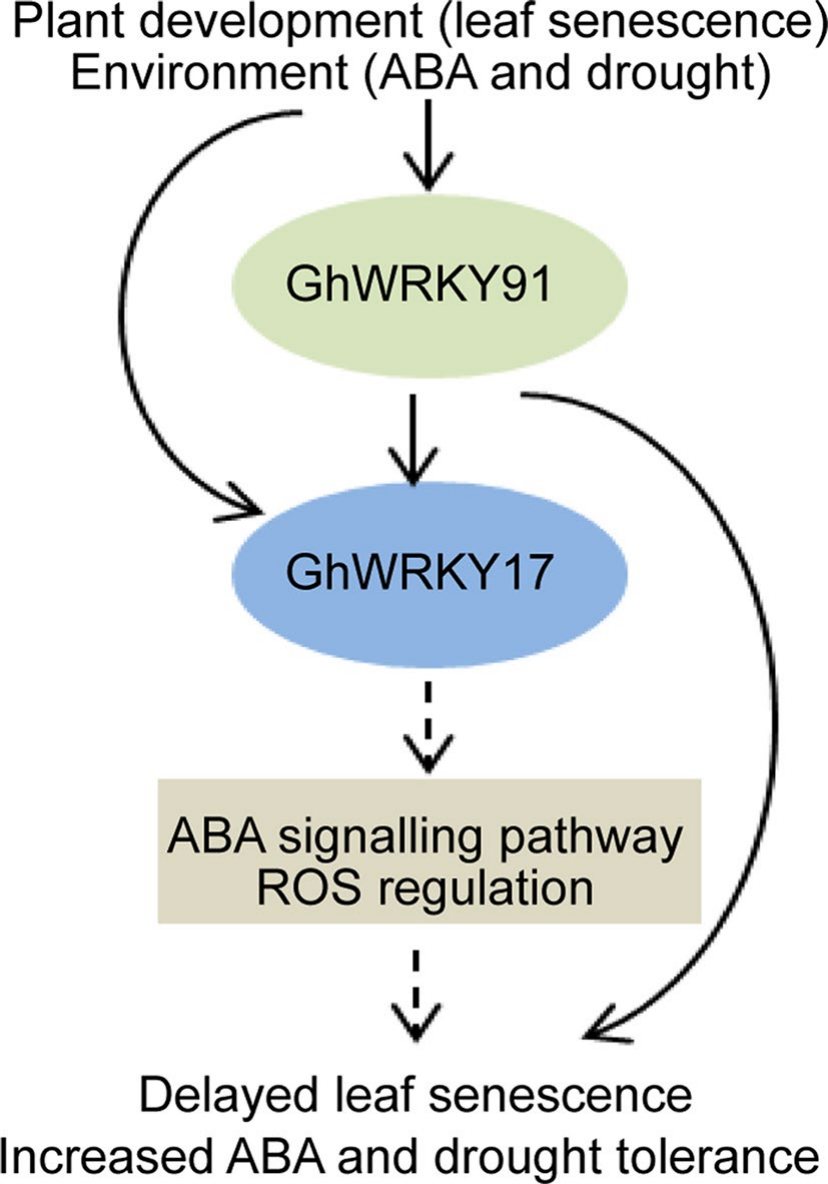
Proposed model for the regulatory mechanism of GhWRKY91. The expression of *GhWRKY91* and *GhWRKY17* is influenced by leaf senescence, ABA, and drought stresses. GhWRKY91 actives the expression of *GhWRKY17*, which is associated with ABA signalling pathways and ROS production. The solid arrows indicate results that have been experimentally determined, whereas dashed arrows indicate speculated effects supported by the literature.

In summary, we assumed that GhWRKY91, which contributes to delayed natural leaf senescence and stress-induced (ABA and drought) leaf senescence in transgenic *Arabidopsis* plants, activates the expression of *GhWRKY17*, a gene associated with ABA signalling pathways and ROS production (**Figure 8**). However, the complex regulatory mechanisms underlying these phenomena remain to be clarified in further studies. Our results provide valuable information for helping understand the relationships among WRKY TFs and increase our understanding of the molecular mechanisms of GhWRKY91 during leaf senescence and stress responses in cotton. In addition, these findings provided a theoretical basis for cultivating cotton varieties with non-premature senescence and stress resistance.

## Data Availability Statement

All datasets for this study are included in the article/ **Supplementary Material**.

## Author Contributions

SY and HTW designed the research program. HTW, LG, and HLW analyzed the data. QM, CZ, and CW revised the language and collected the data. LG performed the experiment and wrote the manuscript. All authors have read and approved the final manuscript.

## Funding

This work was supported by the China Agriculture Research System (grant number CARS-15-06).

## Conflict of Interest

The authors declare that the research was conducted in the absence of any commercial or financial relationships that could be construed as a potential conflict of interest.

## Abbreviations

ABA, abscisic acid; EMSA, electrophoretic mobility shift assay; GUS, β-glucuronidase; qRT-PCR, quantitative real-time PCR; ROS, reactive oxygen species; SAGs, senescence-associated genes; TFs, transcription factors; and WT, wild-type.
