# Supplementary material for: The Cotton GhWRKY91 Transcription Factor Mediates Leaf Senescence and Responses to Drought Stress in Transgenic Arabidopsis thaliana
Source: Front Plant Sci. 2019 Oct 29;10:1352. doi: 10.3389/fpls.2019.01352 (PMC6828947; doi:10.3389/fpls.2019.01352)
Supplement: Supplementary file 1 [file Table_1.docx]

Supplementary Material

## Supplementary Figures


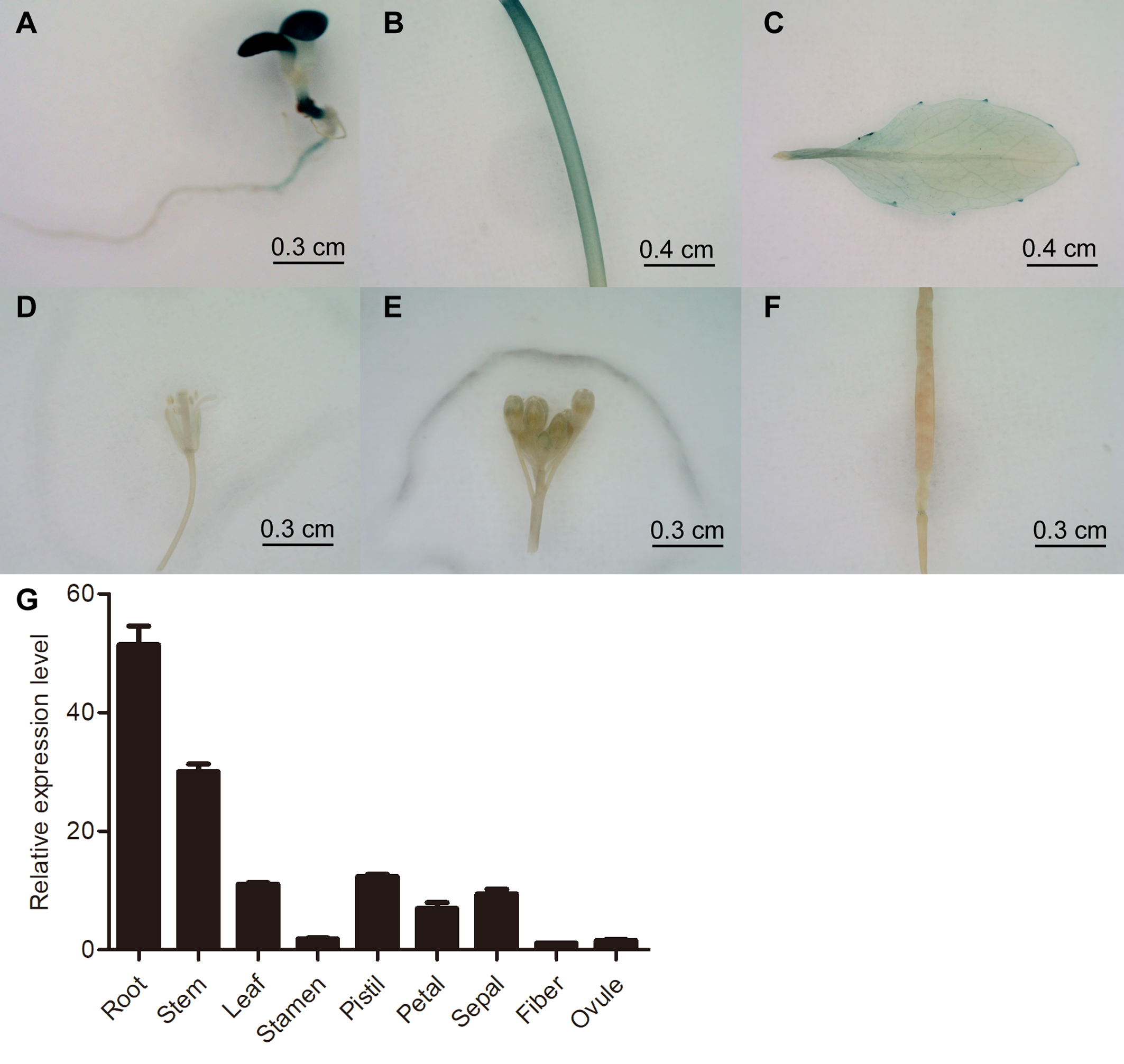


**Supplementary Figure S1.** Expression characteristics of *GhWRKY91* in different tissues. **(A-F)** GUS staining of 8-day-old seedlings as well as stem, leaf, bud, flower and pod tissues of *ProGhWRKY91*::GUS transgenic *Arabidopsis* plants. **(G)** Tissue expression analysis of *GhWRKY91* in the roots, stems, leaves, stamens, pistils, petals, sepals, fibre and ovules of the CCRI10 cotton variety. *GhActin* served as the reference gene. The data are the means ± SEs of three biological replicates.


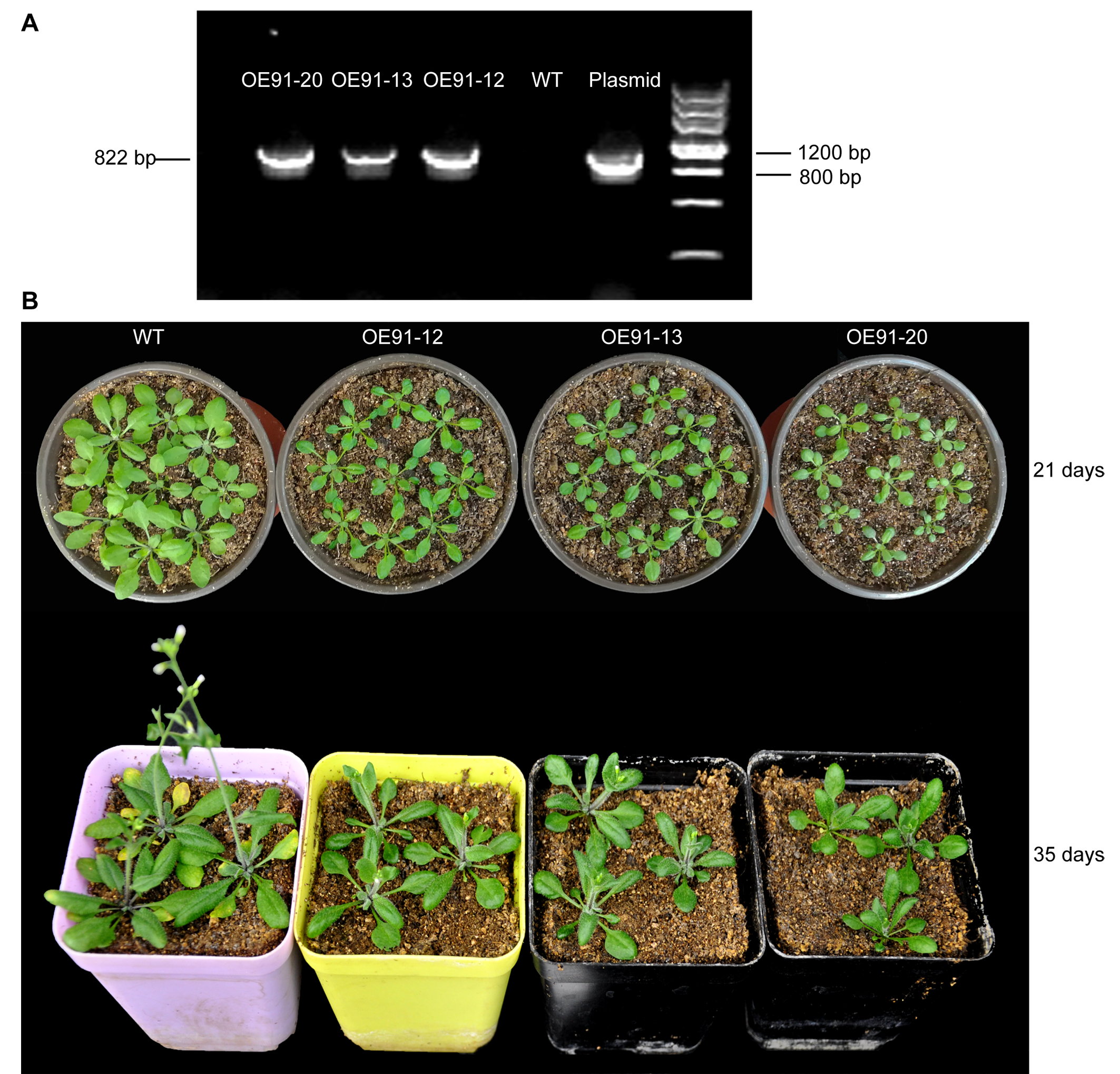


**Supplementary Figure S2.** PCR and phenotypic identification of *GhWRKY91*-overexpressing *Arabidopsis* plants. **(A)** Identification of *GhWRKY91*-overexpressing *Arabidopsis* plants via PCR. The plasmid represents the 35S::*GhWRKY91* recombinant vector. **(B)** Phenotypic characteristics of the WT and transgenic plants grown for 21 and 35 days. The seeds of the WT and transgenic lines were germinated on 1/2MS agar media in a growth chamber at 22°C under a 16 h light/8 h dark photoperiod. Two-week-old seedlings were then transplanted into soil in a greenhouse at 22 ± 1 °C under a 16 h light/8 h dark photoperiod, and the natural growth phenotype was observed for 21-day-old and 35-day-old plants.


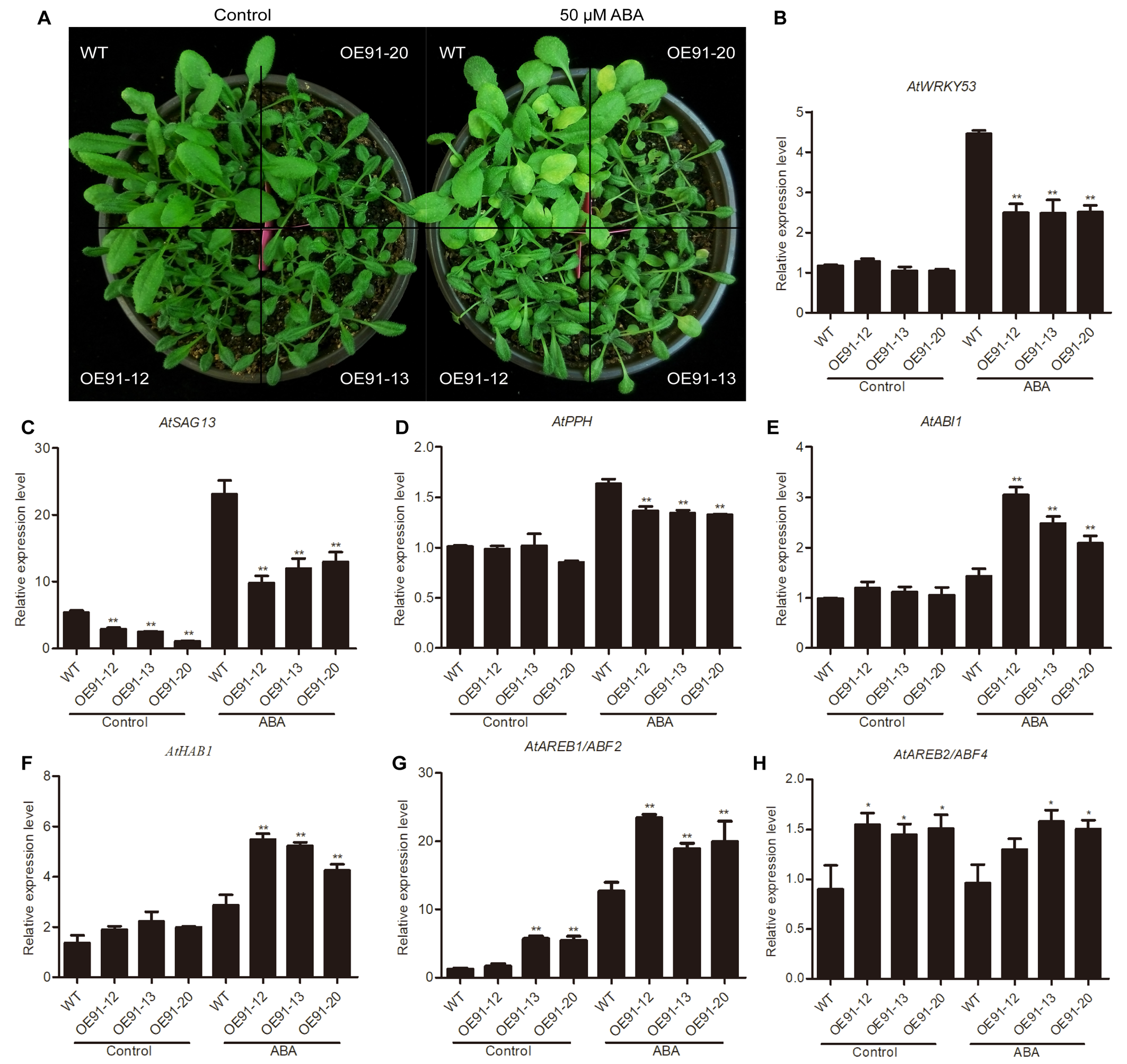


**Supplementary Figure S3.** Overexpression of *GhWRKY91* in *Arabidopsis* delayed ABA-induced leaf senescence. **(A)** Phenotypic characteristics of WT and transgenic plants under control and ABA treatments. Three-week-old WT and transgenic plants grown in soil were sprayed with 50 μM ABA and then were allowed to grow normally for 2 days. Plants grown under normal growth conditions severed as controls. All the plants were grown in a greenhouse at 22 ± 1 °C under a 16 h light/8 h dark photoperiod. **(B-D)** Expression levels of SAGs in WT and transgenic plants. **(E-H)** Expression levels of ABA-responsive genes in WT and transgenic plants. *AtUBQ10* was used as the reference control. The data are the means ± SEs of three biological replicates. ** P<0.01 and * P<0.05.


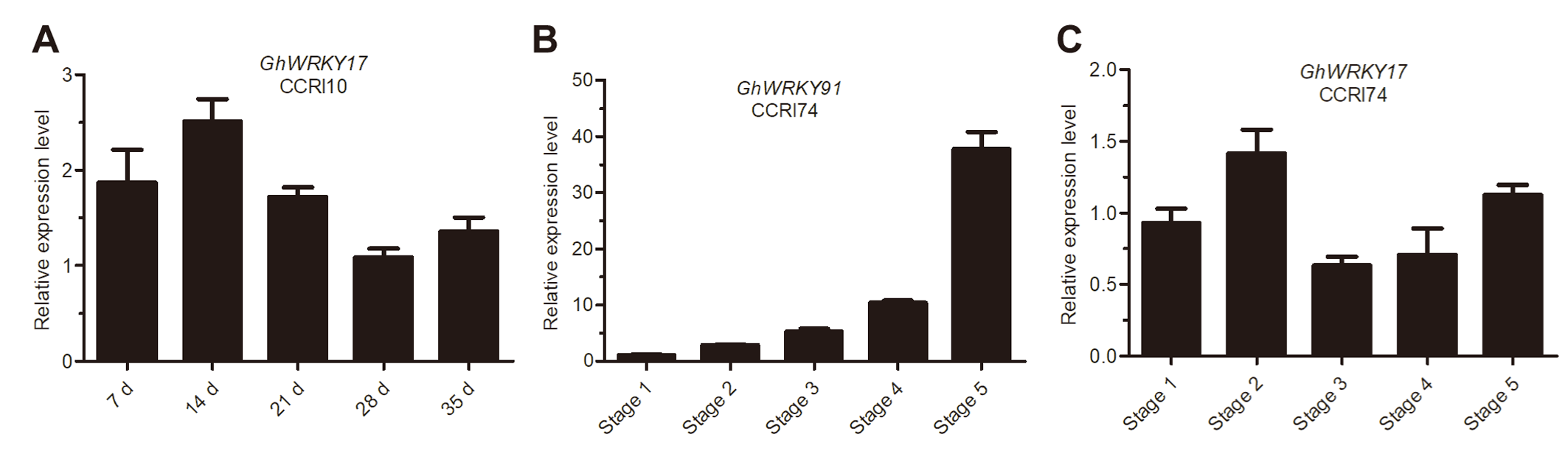


**Supplementary Figure S4.** Expression patterns of *GhWRKY91* and *GhWRKY17* during leaf senescence. **(A)** Expression levels of *GhWRKY17* at different stages of leaf senescence in the CCRI10 variety. **(B-C)** Expression profiles of *GhWRKY91* and *GhWRKY17* in leaves displaying various degrees of leaf senescence. Stages 1-5 represent young leaves, mature leaves without leaf senescence, leaves showing less than 25% leaf senescence, leaves showing approximately 50% leaf senescence and leaves showing more than 75% leaf senescence, respectively. *GhActin* served as the reference gene. The data are the means ± SEs of three biological replicates.

## SupplementaryTables

**Supplementary Table S1. Primers used in this study**

| **Primer name** | **Sequence ( 5’- 3’)** |
| --- | --- |
| gene clone-WRKY91-F | ATGGACAGCGGTGGTAGT |
| gene clone-WRKY91-R | TCAGCAGATCCCAACCT |
| promoter-WRKY91-F | CACGACATCAACATGCACA |
| promoter-WRKY91-R | GGTCGATGATGATGAAGCG |
| qRT-PCR-GhActin-F | ATCCTCCGTCTTGACCTTG |
| qRT-PCR-GhActin-R | TGTCCGTCAGGCAACTCAT |
| qRT-PCR-WRKY91-F | AGACACAACAACACATCCGCT |
| qRT-PCR-WRKY91-R | CCATCCCTGAATCCGGTTCC |
| qRT-PCR-WRKY17-F | GACTGGTGTTTTAGAGCCTGGG |
| qRT-PCR-WRKY17-R | GGAGGTTTGCCATGAGAAGAAGG |
| qRT-PCR-AtUBQ10-F | AGATCCAGGACAAGGAAGGTATTC |
| qRT-PCR-AtUBQ10-R | CGCAGGACCAAGTGAAGAGTAG |
| qRT-PCR-AtHAB1-F | AACTGCTGTTGTTGCCTTG |
| qRT-PCR-AtHAB1-R | GGTTCTGGTCTTGAACTTTCT |
| qRT-PCR-AtABI1-F | ATGATCAGCAGAACAGAGAGT |
| qRT-PCR-AtABI1-R | TCAGTTCAAGGGTTTGCT |
| qRT-PCR-AtAREB1/ABF2-F | CATGATTGGCTGTTGCTGTT |
| qRT-PCR-AtAREB1/ABF2-R | AGGCTCAGAGTCAGAGTCAG |
| qRT-PCR-AtAREB2/ABF4-F | TACTCCCTTACCTTTGATGA |
| qRT-PCR-AtAREB2/ABF4-R | TGTTGCCTTCCAGGAGGCAC |
| qRT-PCR-AtP5CS-F | GAGGGGGTATGACTGCAAAA |
| qRT-PCR-AtP5CS-R | AACAGGAACGCCACCATAAG |
| qRT-PCR-AtP5CS1-F | AAGGCTTGTGATACGGATATGG |
| qRT-PCR-AtP5CS1-R | ATGCACAAGAAGGGTTTCCA |
| qRT-PCR-AtRD29A-F | ATCACTTGGCTCCACTGTTGTTC |
| qRT-PCR-AtRD29A-R | AAAACACACATAAACATCCAAAGT |
| qRT-PCR-AtCOR15A-F | ACTCAGTTCGTCGTCGTTTCT |
| qRT-PCR-AtCOR15A-R | CTTCTTTTCCTTTCTCCTCCAC |
| qRT-PCR-AtSAG12-F | TCCAATTCTATTCGTCTGGTGTGT |
| qRT-PCR-AtSAG12-R | CCACTTTCTCCCCATTTTGTTC |
| qRT-PCR-AtSAG13-F | GTGCCAGAGACGAAACTC |
| qRT-PCR-AtSAG13-R | GCTGTAAACTCTGTGGTC |
| qRT-PCR-AtORE1/ANAC029-F | CTTACCATGGAAGGCTAAGATGGG |
| qRT-PCR- AtORE1/ANAC029-R | TTCCAATAACCGGCTTCTGTCG |
| qRT-PCR-AtWRKY6-F | AGGAAGAACAAGATGATCGAACGGACG |
| qRT-PCR-AtWRKY6-R | TCACCAACTCATTTTTCGCACGCT |
| qRT-PCR-AtWRKY53-F | CAGACGGGGATGCTACGG |
| qRT-PCR-AtWRKY53-R | GGCGAGGCTAATGGTGGT |
| qRT-PCR-AtSGR1-F | TCCAAGAAGAAGAACCAATCGA |
| qRT-PCR-AtSGR1-R | GTATAGCCTATTTGCCCATCCT |
| qRT-PCR-AtPPH-F | CTCCGAAGCTTTATCTAGGTGT |
| qRT-PCR-AtPPH-R | ATCTTCTTTCCCCATAACGGTC |
| qRT-PCR-AtNYC1-F | TGTTATAACCGGAAGCACTAGG |
| qRT-PCR-AtNYC1-R | TTCTTTCTAGCTGACTCGCTAG |
| M13(-47) | TAATACGACTCACTATAGGG |
| 35S | GACGCACAATCCCACTATCC |
| T7 | TAATACGACTCACTATAGGG |
| 3’AD-R | AGATGGTGCACGATGCACAG |
| 3’BD-R | AAGAGTCACTTTAAAATTTGTAT |
| pHIS2-R | TGCCTGCTCATTTTTTAGTATA |
| SK-WRKY91-F(BamHI) | AGAACTAGTGGATCCATGGACAGCGGTGGTAG |
| SK-WRKY91-R(XhoI) | GGGCCCCCCCTCGAGTCAGCAGATCCCAACCT |
| LUC-WRKY17-F(XhoI) | GGGCCCCCCCTCGAGTACTCTTTTATTGGGTCGATAT |
| LUC-WRKY17-R(BamHI) | AGAACTAGTGGATCCCTCTGCTCATACGAAAACAAA |

**Supplementary Table S2. Nucleic acid sequences of the potential target genes of GhWRKY91 used for the Y1H system**

| **Gene ID** | **Gene Name** | **Nucleic acid sequences** |
| --- | --- | --- |
| Gh_A09G0118 | *GhWRKY3* | ATATTATTATTGCTGTTATAATAATTGCATTGACTAATAAAATTCTTAAACCAAATATTATTATTGCTGTTATAATAATTGCATTGACTAATAAAATTCTTAAACCAAATATTATTATTGCTGTTATAATAATTGCATTGACTAATAAAATTCTTAAACCAA |
| Gh_A06G0179 | *GhWRKY17* | AATTTCAAACGGCTAATCCCCACCTTTTGACCTTATCTCTTGTCAGTGACCCAAATTTCAAACGGCTAATCCCCACCTTTTGACCTTATCTCTTGTCAGTGACCCAAATTTCAAACGGCTAATCCCCACCTTTTGACCTTATCTCTTGTCAGTGACCCA |
| Gh_A05G3186 | *GhWRKY25* | ATATACAAATTTATTATTAGCATGATTGACTAATTTAACAATAGTGTCTCTAACAATATACAAATTTATTATTAGCATGATTGACTAATTTAACAATAGTGTCTCTAACAATATACAAATTTATTATTAGCATGATTGACTAATTTAACAATAGTGTCTCTAACA |
| Gh_A08G2417 | *GhWRKY27a* | GCGACTTGGTCAGCAATTGGAGTTTTGACCTTACCTCACTCTTCAGTCCACGCGACTTGGTCAGCAATTGGAGTTTTGACCTTACCTCACTCTTCAGTCCACGCGACTTGGTCAGCAATTGGAGTTTTGACCTTACCTCACTCTTCAGTCCAC |
| Gh_Sca005564G01 | *GhWRKY68* | GCACTGTTCAATATCCGTACAAGTGGTTGACCTACAATTGCCCGTCGATCCGCACTGTTCAATATCCGTACAAGTGGTTGACCTACAATTGCCCGTCGATCCGCACTGTTCAATATCCGTACAAGTGGTTGACCTACAATTGCCCGTCGATCC |
| Gh_A05G0863 | *GhAPX1* | TCCCCGACGTTCCGACAAGTTTTGACTATTTTATTTTCTTAAAATAAAAAGCTCCCCGACGTTCCGACAAGTTTTGACTATTTTATTTTCTTAAAATAAAAAGCTCCCCGACGTTCCGACAAGTTTTGACTATTTTATTTTCTTAAAATAAAAAGC |
| Gh_D05G0507 | *GhRD22* | AACACCAAAAGTTTAATATCTAGATTTTGACCATATTTACGTTAACAAAGTAAAACACCAAAAGTTTAATATCTAGATTTTGACCATATTTACGTTAACAAAGTAAAACACCAAAAGTTTAATATCTAGATTTTGACCATATTTACGTTAACAAAGTAA |

Red indicates the W-box cis-element.

**Supplementary Table S3. Predicted *cis*-acting elements in the promoter of *GhWRKY91***

| **Cis-element** | **Position** | **Sequence (5’-3’)** | **Function** |
| --- | --- | --- | --- |
| ***Stress responsive elements*** | | | |
| ABRE | -1576(-) | ACGTG | Cis-acting element involved in the ABA responsiveness |
| ARE | -1618(+) | AAACCA | Cis-acting regulatory element essential for the anaerobic induction |
| LTR | -124(+) | CCGAAA | Cis-acting element involved in low-temperature responsiveness |
| ***Light responsive elements*** | | | |
| AE-box | -1683(-) | AGAAACAA | Part of a module for light response |
| Box 4 | -116(+) | ATTAAT | Part of a conserved DNA module involved in light responsiveness |
| I-box | -115(+) | GTATAAGGCC | Part of a light responsive element |
| G-box | -1576(+) | CACGTC | Cis-acting regulatory element involved in light responsiveness |
| GT1-motif | -1134(+) | GGTTAA | Light responsive element |
| Sp1 | -1650(+) | GGGCGG | Light responsive element |
| ***Development-related elements*** | | | |
| O2-site | -1754(-) | GATGATGTGG | Cis-acting regulatory element involved in zein metabolism regulation |
| CAT-box | -269(-) | GCCACT | Cis-acting regulatory element related to meristem expression |
| ***Others*** | | | |
| AT-rich element | -970(-) | ATAGAAATCAA | Binding site of AT-rich DNA binding protein (ATBP-1) |
| MBSI | -824(-) | TTTTTACGGTTA | MYB binding site involved in flavonoid biosynthetic genes regulation |
| W-box | -758(+) | TTGACC |  |
